# Supplementary material for: Human DNA polymerase delta requires an iron–sulfur cluster for high-fidelity DNA synthesis
Source: Life Sci Alliance. 2019 Jul 5;2(4):e201900321. doi: 10.26508/lsa.201900321 (PMC6613617; doi:10.26508/lsa.201900321)
Supplement: Supplementary file 1 [file LSA-2019-00321_TableS1.doc]

**Table S1. Error rates of exonuclease-deficient (WT exo –) or exonuclease-proficient (WT exo ^+^) Pol δ in the absence of PCNA in a pSJ4-*lacZα* forward mutation assay.**

| **Pol δ** | **Total number of colonies ^a^** | **Number of white mutants** | **Corrected**  **mutant**  **frequency ^b^** | **Error**  **rate ^c^** |
| --- | --- | --- | --- | --- |
| *WT exo ­^–^* | 27,113 | 25 | 7.1 x 10^-4^ | 1.6 x 10^-5^ |
| *WT exo ^+^* | 24,035 | 12 | 2.9 x 10^-4^ | 6.6 x 10^-6^ |

**^a^** The fidelity of each polymerase variant was determined in three separate experiments. The aggregated numbers are given. **^b^** Mutant frequency equals: (number of white colonies/ total number of colonies) – background mutant frequency. A background mutant frequency of 4.8 x 10^-6^ was used for gapped pSJ4. **^c^** Error rate is the number of mistakes made per base incorporated. The corrected mutant frequency was converted to error rate as previously described (Keith et al., 2013). An expression frequency (P) of 0.3 was used. Due to the limited amount of sequencing data, a set Ni/N value of 1 was used and the number of detectable sites (D) was the sum of the determined base substitutions plus insertions/ deletions, that is 145 in pSJ4.
