# Supplementary material for: Human DNA polymerase delta requires an iron–sulfur cluster for high-fidelity DNA synthesis
Source: Life Sci Alliance. 2019 Jul 5;2(4):e201900321. doi: 10.26508/lsa.201900321 (PMC6613617; doi:10.26508/lsa.201900321)
Supplement: Supplementary file 2 [file LSA-2019-00321_TableS2.doc]

**Table S2. Mutation spectra of different Pol δ variants in the presence of PCNA in a pSJ4-*lacZα* forward mutation assay.**

| **Mutation type** | **Pol δ + PCNA** | | | | | | | | | |
| --- | --- | --- | --- | --- | --- | --- | --- | --- | --- | --- |
|  | *WT exo –* | | *WT exo ^+^* | | *CS exo ^+^* | | *HY exo ^+^* | | *HW exo ^+^* | |
|  | Number | Frequency (%) | Number | Frequency (%) | Number | Frequency (%) | Number | Frequency (%) | Number | Frequency (%) |
| A>T/T>A | 10 | 12 | 3 | 8 | 13 | 18 | 13 | 19 | 6 | 6 |
| A>C/T>G |  |  |  |  |  |  | 4 | 6 |  |  |
| A>G/T>C | 7 | 8 | 2 | 5 | 2 | 3 | 1 | 1 | 3 | 3 |
| G>A/C>T | 31 | 36 | 22 | 55 | 37 | 51 | 25 | 37 | 42 | 43 |
| G>C/C>G |  |  |  |  |  |  | 4 | 6 |  |  |
| G>T/C>A | 13 | 15 |  |  | 1 | 1 | 3 | 4 | 15 | 15 |
| Single nt insertion | 1 | 1 |  |  | 1 | 1 | 1 | 1 |  |  |
| Single nt deletions | 19 | 22 | 12 | 30 | 14 | 19 | 14 | 21 | 29 | 30 |
| Multiple deletions | 2 | 2 | 1 | 3 | 1 | 1 |  |  |  |  |
| Mixed | 2 | 2 |  |  | 3 | 4 | 3 | 4 | 2 | 2 |
| **Total** | **85** | **100** | **40** | **100** | **72** | **100** | **68** | **100** | **97** | **100** |
| A>N/T>N | 17 | 20 | 5 | 13 | 15 | 21 | 18 | 26 | 9 | 9 |
| G>N/C>N | 44 | 52 | 22 | 55 | 38 | 53 | 32 | 47 | 57 | 59 |
| Transitions | 38 | 45 | 24 | 60 | 39 | 54 | 26 | 38 | 45 | 46 |
| Transversions | 23 | 27 | 3 | 8 | 14 | 19 | 24 | 35 | 21 | 22 |
| Deletions/ insertions | 24 | 28 | 13 | 33 | 19 | 26 | 18 | 26 | 31 | 32 |

nt: nucleotide.
