# Supplementary material for: Human DNA polymerase delta requires an iron–sulfur cluster for high-fidelity DNA synthesis
Source: Life Sci Alliance. 2019 Jul 5;2(4):e201900321. doi: 10.26508/lsa.201900321 (PMC6613617; doi:10.26508/lsa.201900321)
Supplement: Supplementary file 4 [file LSA-2019-00321_TableS4.doc]

**Table S4. Sequencing primers used in the study.**

| **Name** | **Primer (5´­–3´)** |
| --- | --- |
| POLD1-sRP1 | TGCCATGGCCCTTCCGGTGGGTG |
| POLD1-sFP1 | CATCCCGCGGCTCCGTGCCTGTG |
| POLD1-sFP2 | AGCTGGGAAATACGCCCTGAG |
| POLD1-sFP3 | GAAGGAGGACGTGCAGCACAGCATC |
| POLD1-sFP4 | GCTGAAGGTGAGCGCCAACTCCG |
| POLD1-sFP5 | ATGAGGAAGCGGACCCCGGGAGTG |
| pFASTbac1-sFP1 | GTTTTCCCAGTCACGACGTTGTAAAACG |
| pFASTbac1-sRP1 | CAGGAAACAGCTATGACCATGATTACG |
| pSJ4-sFP1 | ATGCAGCTGGCACGACAGGTTTC |
