# Supplementary material for: Human DNA polymerase delta requires an iron–sulfur cluster for high-fidelity DNA synthesis
Source: Life Sci Alliance. 2019 Jul 5;2(4):e201900321. doi: 10.26508/lsa.201900321 (PMC6613617; doi:10.26508/lsa.201900321)
Supplement: Supplementary file 5 [file LSA-2019-00321_TableS5.doc]

**Table S5. Sequences of oligonucleotides used for the generation of DNA substrates.**

| **Figure** | **Labelled top strand (5´–3´)** | **Unmodified bottom strand (5´–3´)** |
| --- | --- | --- |
| 2C,D  4B,C,E,F  6B  S2C  S3B  S6A–D | **FAM–**GACGCTGCCGAATTCTACCAG TGCCTTGCTAGGACATCTTTG | GGGTGAACCTGCAGGTGGGCAAAGATGTCCTAGCAAGGCACTGGTAGAATTCGGCAGCGTC |
| 3A,B | **FAM­–**GTTTCTTCAATCTTCATCA  TACAGGTCAG | CTCGTCAGCATCTCGTCTGACCTGTATGATGAAGATTGAAAGAAAC |
| 3C,D | **FAM–**GTTTCTTCAATCTTCATCA  TACAGGTCAG | CTGACCTGTATGATGAAGATTGAAAGAAAC |
| 3E,F | **FAM–**GTTTCTTCAATCTTCATCA  TACAGGTCAG | **–** |

FAM indicates 5´-fluorescein amidite label.
