# Supplementary material for: Human DNA polymerase delta requires an iron–sulfur cluster for high-fidelity DNA synthesis
Source: Life Sci Alliance. 2019 Jul 5;2(4):e201900321. doi: 10.26508/lsa.201900321 (PMC6613617; doi:10.26508/lsa.201900321)
Supplement: Supplementary file 6 [file LSA-2019-00321_TableS6.doc]

**Table S6. Single-stranded DNA competitors used in the study.**

| **Name** | **Primer (5´–3´)** |
| --- | --- |
| T61 competitor | GACGCTGCCGAATTCTACCAGTGCCTTGCTAGGACATCTTTGCCCACCTGCAGGTTCACCC |
| pSJ4 competitor | GGTTTTCCCAGTCACGACGTTGTAAAACGACGGCCAGTGAATTCGTAATCATGGTCATAGCTGA |
